# Three-Dimensional Analysis of the First Metacarpal Axes in Healthy Individuals and Early-Stage Thumb Carpometacarpal Osteoarthritis Patients—Potential Implication on First Metacarpal Corrective Osteotomy

**DOI:** 10.3390/jcm13185513

**Published:** 2024-09-18

**Authors:** Marco Keller, Jasmine Rueegg, Mathias Haefeli, Philipp Honigmann

**Affiliations:** 1 Hand and Peripheral Nerve Surgery, Department of Orthopaedic Surgery and Traumatology, Kantonsspital Baselland (Bruderholz, Liestal, Laufen), 4101 Bruderholz, Switzerland; philipp.honigmann@ksbl.ch; 2 Hand and Peripheral Nerve Surgery, Department of Orthopaedic Surgery, Traumatology and Hand Surgery, Spital Limmattal, 8952 Schlieren, Switzerland; 3 Medical Additive Manufacturing Research Group (MAM), Department of Biomedical Engineering, University of Basel, 4123 Allschwil, Switzerland; 4 Department of Orthopaedic and Trauma Surgery, Ninewells Hospital and Medical School, University of Dundee, Dundee DD1 4HN, UK; 5 Clinic of Hand Surgery, Kantonsspital Graubünden, 7000 Chur, Switzerland; mathias.haefeli@bluewin.ch; 6 Department of Biomedical Engineering and Physics, Amsterdam UMC, University of Amsterdam, Amsterdam Movement Sciences, 1105 Amsterdam, The Netherlands

**Keywords:** first metacarpal axis, (Wilson) extension osteotomy, trapeziometacarpal joint, first carpometacarpal joint, ligamentoplasty, osteoarthritis

## Abstract

**Background:** Numerous anatomical features of the first carpometacarpal (CMC I) joint have been investigated as potential predispositions for CMC I osteoarthritis (OA). Even though load transmission through the CMC I joint—and, therefore, the development of osteoarthritis—is believed to be influenced by the geometry of the first metacarpal (MC I) bone, there is no common definition of the MC I axes. **Methods:** CT scans of twenty healthy volunteers and pre- and postoperative CT scans of six patients with CMC I OA undergoing Wilson osteotomy were analyzed. We proposed a calculation method based on anatomical landmarks for the proximal joint surface axis (PA) angle and the definition of an anatomical (AA) and a mechanical (MA) longitudinal axis. We hypothesized that for an MC I extension osteotomy to be effective, the AA and MA need to be aligned surgically. **Results**: To align AA and MA, an average correction angle of 22.60° (SD 2.53°) at 1 cm and 26.73° (SD 2.55°) at 1.5 cm distal to the CMC I joint line is required. **Conclusions:** The hereby proposed method for patient-specific calculation of the correction can be used to improve the surgical technique.

## 1. Introduction

Numerous anatomical features of the first carpometacarpal (CMC I) joint have been investigated as potential predispositions for CMC I laxity and therefore osteoarthritis (OA) [1]. Most research focusses on the morphology of the articular surface. However, load transmission through the CMC I joint is also influenced by the more distal aspects of the first metacarpal bone (e.g., shaft curvature and center of rotation of the head). Therefore, the entire geometry of the first metacarpal bone (MC I) should be considered while investigating contributing factors to CMC I OA. Research about the possible impact of the angle between the longitudinal axis of MC I in relation to its proximal articular surface (first metacarpal tilt angle) is still scarce [2,3,4,5]. Although the first metacarpal tilt angle is described in some studies, it is not uniformly defined.

**Figure 1:**
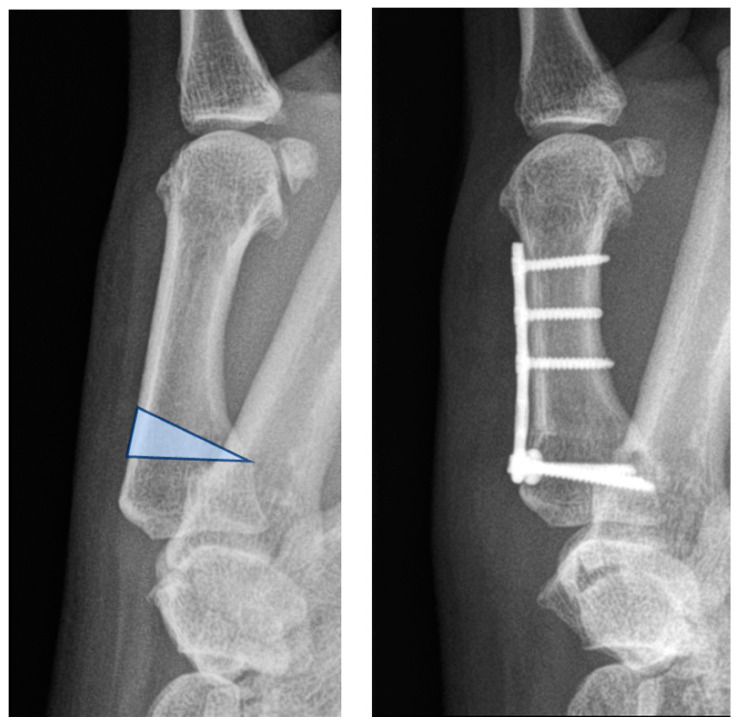
Wilson osteotomy of the first metacarpal bone. The blue triangle highlights the bone section which is excised during the closing-wedge osteotomy.

The potential influence of the first metacarpal tilt angle serves as rationale for MC I extension osteotomy. The procedure was described for early-stage CMC I OA by Wilson et al. in 1973 and shows very satisfying short- and long-term results [6,7,8,9]. The concept behind the procedure is to re-center the metacarpal bone over the trapezium so that load transmission is directed to the healthy part of the joint. To achieve this effect, a closing wedge extension osteotomy of around 20–30° in the region 5–10 mm distal to the articular surface is proposed (Figure 1), yet no precise calculation of the correction angle has been described, nor could it be fully explained why this intervention provides relief [6]. To increase joint stability, the procedure is frequently combined with a ligamentoplasty, i.e., the technique developed by Brunelli [10,11].

Studies about the first metacarpal tilt angle of healthy and osteoarthritic CMC I joints usually suggest calculations based on two-dimensional lateral plain radiographs. The aim of this study was to define the necessary axes of the first metacarpal bone based on three-dimensional (3D) image data to reliably calculate the first metacarpal tilt angle, compare the accuracy of the 3D-based calculation with conventional 2D-based methods and to determine the correction angle to be used for first metacarpal extension osteotomy.

## 2. Material and Methods

### 2.1. Image Acquisition and Processing

Computed tomography (CT) scans of 20 healthy volunteers consisting of 10 males and 10 females with a mean age of 32.3 years were used to establish the measurement method of the MC-I axes (Appendix A: CT acquisition settings). Additionally, six CT scans of patients with symptomatic early-stage CMC I OA undergoing extension osteotomy of the first metacarpal bone were available. All six patients were female, with a mean age of 44.5 years. Preoperative CT-scans were available for all six, and postoperative CT-scans were available for four of these patients. The CT images underwent semi-automated segmentation using Mimics (v.24, Materialise, Leuven, Belgium) to acquire 3D models of the first metacarpal and trapezium. For 16 of the 20 healthy volunteers and all 6 of the CMC1 OA patients, additional two-dimensional (2D) plain radiographs were available.

All image data used in this study were obtained with written informed consent for use. Ethical approval for the acquisition and use of the image data was granted by the Ethics Committee of Northwestern and Central Switzerland (EKNZ number 2020-02199).

### 2.2. First Metacarpal Bone

**Measurement.** All measurements of the 3D data were performed on each MC I manually using Geomagic Freeform (GFP) (v.2021.0.56, 3D Systems) software. The measurements of the 2D data were performed with Autodesk Inventor Professional (v.2018, Autodesk). Alterations of orientation and size were completed once manually with the GFP tool and once with a Python program using the “trimesh” library (v.3.2.0) [12]. This resulted in two datasets with the same source data. Measurements were taken for both datasets as an intraobserver study.

**Figure 2:**
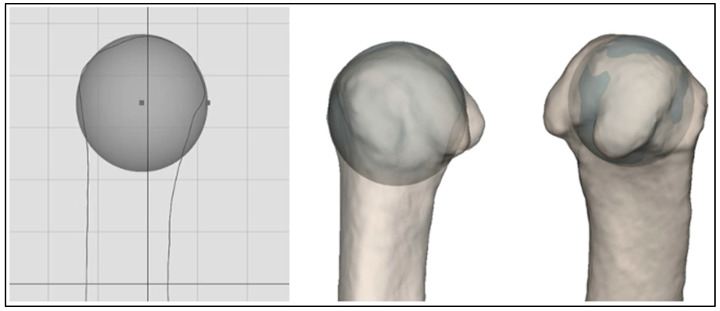
Distal anchor point of the mechanical axis of MC I: center of the sphere created on the MC-1 head.

**Metacarpal I axis.** Regarding the longitudinal MC I axis, an anatomical (AA) and a mechanical axis (MA) were defined; the anatomical axis is the inertia axis of the bone (basically the axis of rotation, which is clearly defined and can be calculated automatically), and the mechanical axis is calculated as follows: the intersection of MC I at the mirror plane is projected onto the plane and a circle is drawn on the MC I head. This circle provides the diameter for the sphere to be created, which is mapped on to the MC I head (Figure 2). The *distal anchor point of the mechanical axis* is the center of this sphere. This technique is based on the method for the definition of joint coordinate systems recommended by the International Society of Biomechanics (ISB) [13].

**Figure 3:**
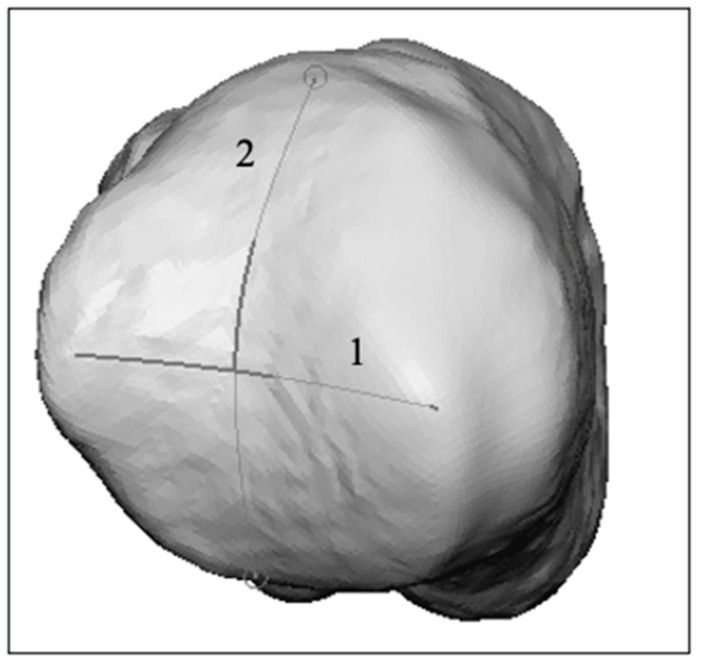
Two curves drawn in the biconcave joint of the MCI joint whose intersection is the proximal anchor point of the mechanical axis of MC I.

The *proximal anchor point of the mechanical axis* is the intersection between the two motion arcs of the proximal biconcave joint surface of the MC I [1]. The first curve (curve 1 in Figure 3) runs from the dorsal to the most volar aspect, whereas the most proximal point of the dorsal rim is connected to the most proximal point of the volar rim via the most distal point of the medial articulating surface. The second curve (curve 2 in Figure 3) runs from ulnar to radial, whereas the most distal points are selected at the outer edges running via the most proximal point on the medial articulating surface (Figure 3).

**Figure 4:**
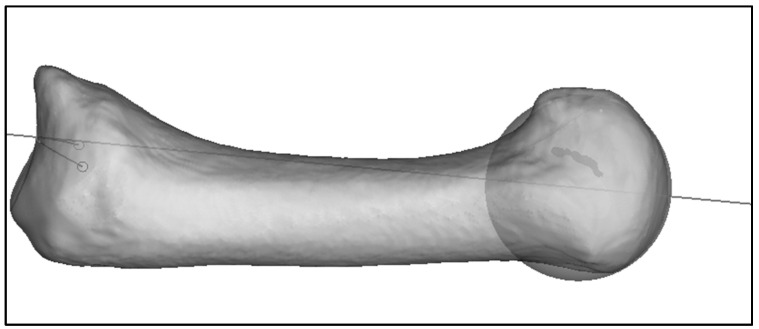
Mechanical axis of MC I.

The proximal and distal anchor points of the mechanical axis are connected and tangentially extended (Figure 4).

**Figure 5:**
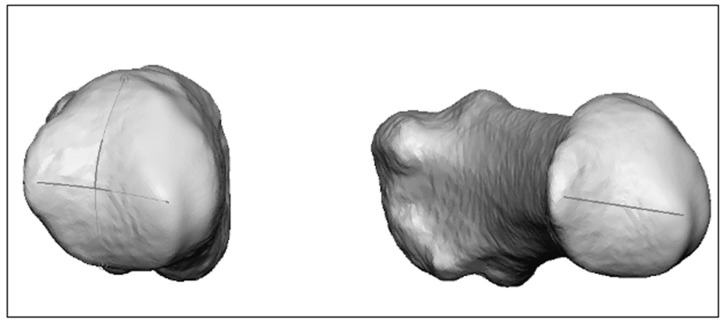
Method of determining the proximal MC I joint surface axis.

**Proximal MC I joint surface axis.** The proximal joint surface axis (PA) of the MC I is estimated with a slight adaption to Rusli et al., where the most proximal points of the articulating surface are connected and not only a straight line from the dorsal to the volar rim is used (Figure 5) [2]. This axis represents the dorsal–volar curve of the two curves which were used to estimate the proximal anchor point of the mechanical axis (curve 1 in Figure 3).

**Figure 6:**
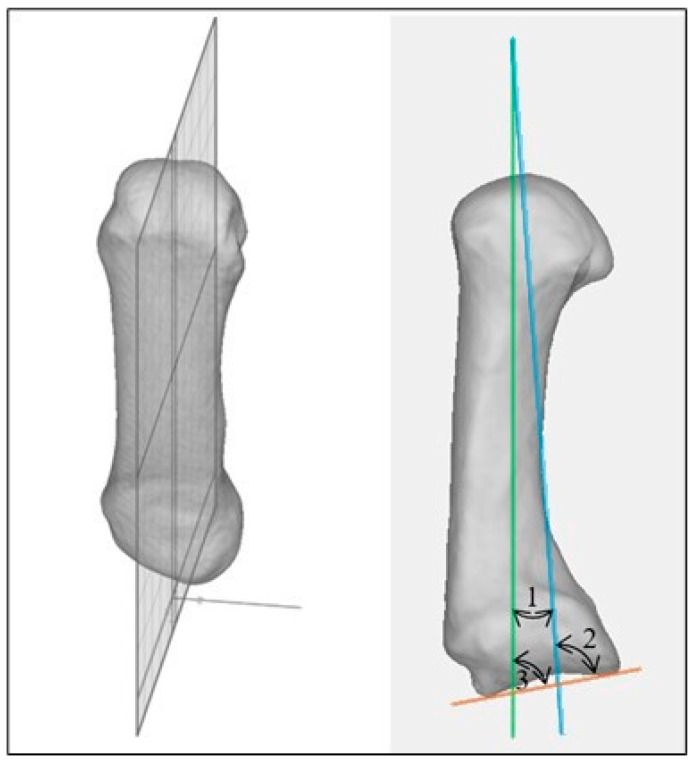
(**Left**) mirror plane as a coronal cross-section; (**right**) measured angles on plane. Anatomical axis of MC I = green; mechanical axis of MC I = blue; proximal joint axis of MC I = orange.

**Angle measurement.** All angles were measured in a coronal cross-section along the inertia axis, in the approximate middle of the MC I. Therefore, the mechanical and the proximal joint axis were projected onto this plane as shown in Figure 6 and the following angles were measured:

Anatomical axis–mechanical axis angle (number 1 in Figure 6).

*Anatomical axis–proximal joint axis angle* (=metacarpal tilt angle, anatomically) (number 3 in Figure 6; the angle was measured on the volar side in the coronal view).

*Mechanical axis–proximal joint axis angle* (=metacarpal tilt angle, mechanically) (number 2 in Figure 6; the angle was measured on the volar side in the coronal view).

**Comparison of exit points of MC I axes.** All analyzed MC I were registered to each other, so that the anatomical and the mechanical axes give an exit area at the proximal and distal end of the MC I. The registration of the bones was conducted with the Python program and manually with the GFP tool, where one MC I was defined as the master and all the other MC I were registered to this.

### 2.3. Wilson Osteotomy Correction Angle Calculation

All angles of the six CMC I OA patients were calculated according to the method described in Section 2. In addition, the correction angle for the four datasets with postoperative data was calculated and compared with the actual performed correction angle. We hypothesize that for an MC I extension osteotomy to be effective, the anatomical axis (AA) and mechanical axis (MA) need to be aligned surgically. This leads to the re-centering of the metacarpal bone over the trapezium so that load transmission is directed to the healthy part of the joint. This is believed to eventually lead to the relief of the patient’s symptoms.

**Figure 7:**
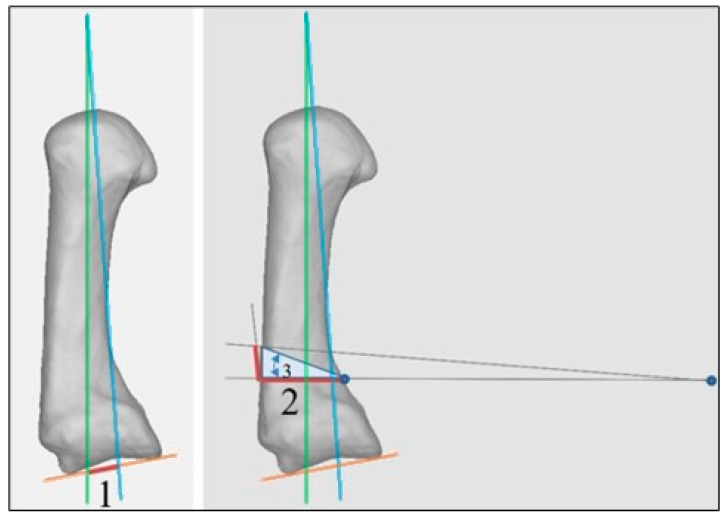
Visualization of the correction angle calculation.

To approximate the anatomical axis to the mechanical axis, a closing-wedge extension osteotomy according to Wilson is performed [7]. The bone wedge that needs to be removed is calculated as follows: the center of rotation for the correction wedge (blue point in Figure 7) is not the same as the center of rotation for the approximation of the two axes. Therefore, a simplification is made: the triangle of the anatomical (green), mechanical (blue) and proximal joint line axes (yellow) is aligned with the first proximal osteotomy cutting plane (horizontal to the AA). The width of the bone at the correction site must be taken into account, since the osteotomy has to end and the palmar cortex. This distance (1) is now projected on to the dorsal cortex and highlights the height of the second, distal osteotomy site, aiming towards the blue point. This will lead to the intended correction (which is aligning AA and MA). The excision of the bone wedge could be conducted under fluoroscopy control or, for more precision, using patient-specific osteotomy guides.

**Figure 8:**
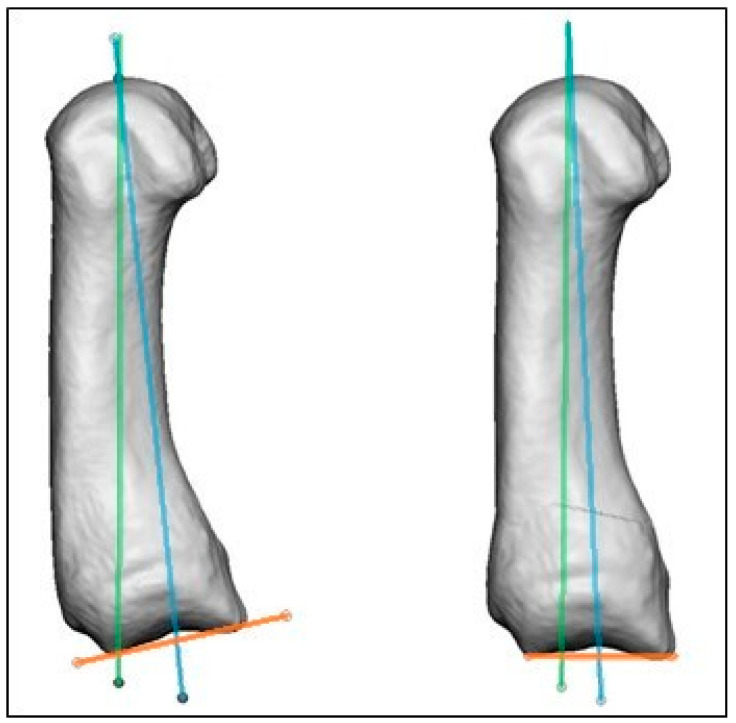
(**Left**) original MC I; (**right**) MC I after Wilson osteotomy simulation with a 90° angle between AA and PA.

**Wilson osteotomy simulation.** In order to calculate correction angles for hypothetical Wilson osteotomies in the healthy volunteers’ MC I, the osteotomy was simulated with the objective to bring the anatomical axis to the proximal joint axis at 90°. This is a commonly used approach to plan the correction angle preoperatively. The simulated osteotomy was performed 1 cm distal to the joint surface and is shown in Figure 8. 

### 2.4. Comparison between 3D and 2D Data

**Figure 9:**
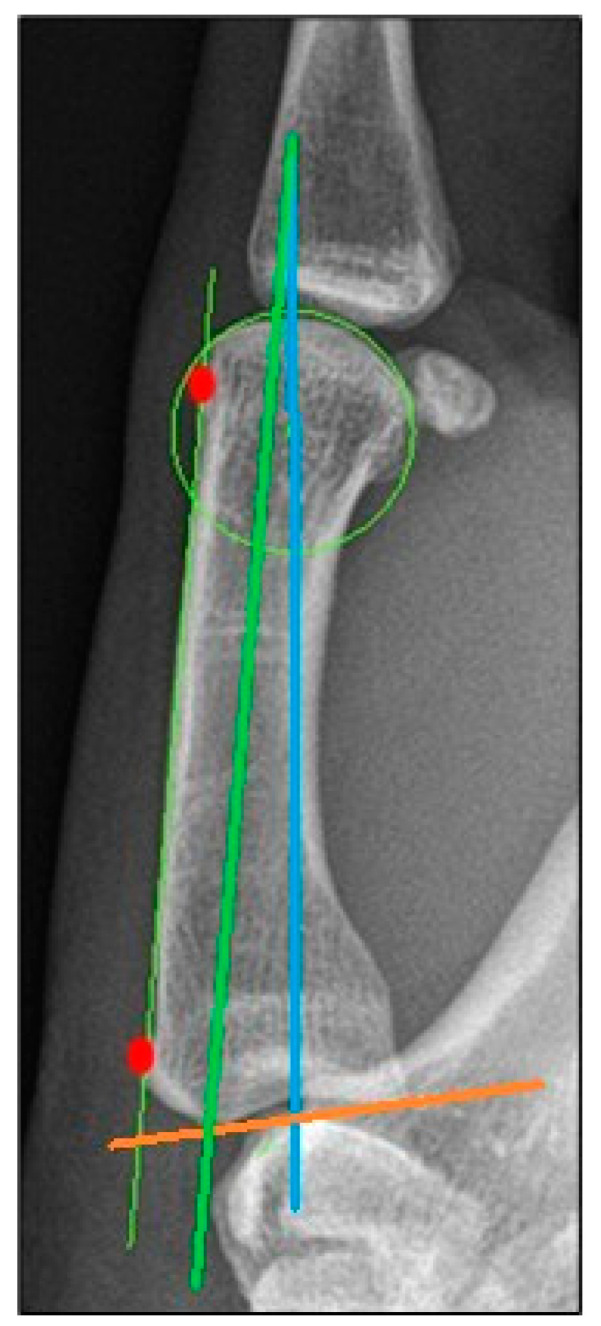
Determination of the axes in lateral conventional radiographs.

Since 3D data are rarely available in CMC I OA patients, we conducted a comparison between 2D and 3D data. The axes in lateral conventional radiographs were calculated as follows:

*Anatomical axis*: midline of the MC I parallel to the dorsal cortex. The axis is defined with the most dorsal point just below the MC I head and the most dorsal point just above the MC I base (red dots in Figure 9).

*Mechanical axis*: the connection between the center of a circle which encloses the MC I head and the most distal point of the proximal articular surface (Figure 9).

*Proximal joint axis*: the two most proximal points connected to each other (Figure 9).

### 2.5. Intraobserver Study

In order to test the feasibility of the measurement method described here, all measurements of the healthy data were carried out twice (by the same user) as an intraobserver study. The data were prepared once manually with GFP and once automatically with the Python program. The datasets from the two measurements were then compared and the absolute and relative difference, the absolute and relative individual standard deviation of each measured value and the average values were determined. The intraclass correlation coefficient (ICC) was used to reflect the variation in data measured by one user for the two datasets. A value close to one indicates high reliability, and a value close to zero low reliability [14,15].

## 3. Results

### 3.1. Metacarpal I Measurements in Healthy Volunteers

**Figure 10:**
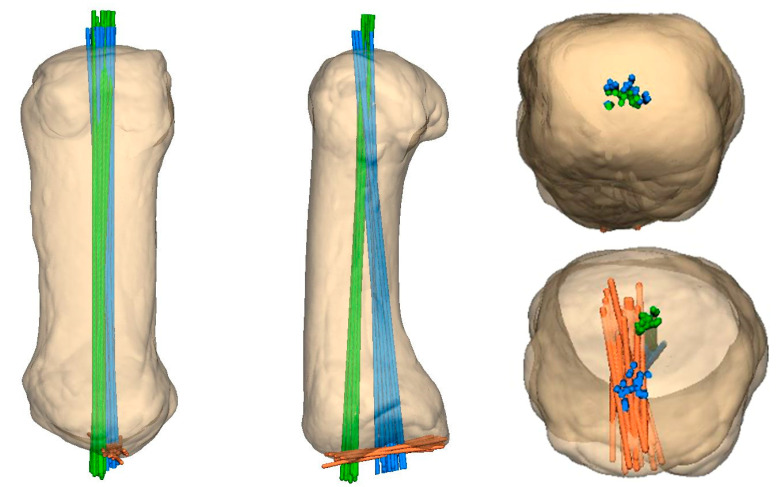
Anatomical axis of MC I combined = green; mechanical axis of MC I combined = blue; proximal joint axis of MC I combined = orange.

All three defined axes (AA, MA, PA) of the 20 healthy individuals are visualized with separate colors within the normalized and equally aligned MC I bones (Figure 10).

The average angle between the proximal joint axis and the mechanical axis (first metacarpal tilt angle, mechanically) is 85.40°, with an SD of 2.88°. The average angle between the proximal joint axis and the anatomical axis (first metacarpal tilt angle, anatomically) is 79.35°, with an SD of 3.18°. The average angle between the anatomical and the mechanical axis is 6.09°, with an SD of 1.22°. The mechanical axis has a mean angle of 1.45°, with an SD of 0.86°, to the anatomical axis in the dorsal view. The proximal joint axis is 9.63° angulated to the middle plane of the MC I with a rather greater SD of 5.40° (Table 1).

### 3.2. Metacarpal I Measurements in Symptomatic Early-Stage Osteoarthritis Patients

Measurements of the four symptomatic datasets resulted in a preoperative mean first metacarpal tilt angle of 84.79° (mechanically, SD 3.40°) and 78.88° (anatomically, SD 3.86°), which are approximately same values as in the healthy volunteer dataset (Table 2). The metacarpal tilt angle for both longitudinal axes (mechanical and anatomical) was corrected to a mean postoperative value of approximately 107° (Table 3). For the six symptomatic MC I bones, the mean calculated correction angle at 1 cm above the proximal joint surface was 22.45° (SD 3.07°). “Calculated”, in this context, means that this is the correction angle which would be necessary to align the AA and the MA. For the four actually surgically corrected MC I, the approximation of the two longitudinal axes is visible with a small angulation remaining between them. The calculated average correction angle (22.45°) was smaller than the actually executed average correction angle (28.68°), which means that in vivo, a slight overcorrection was conducted (Table 4).

### 3.3. Wilson Osteotomy Correction Angle Calculation in the Healthy Volunteer Dataset

Based on the analysis of the healthy MC I bones, bone thickness at 1 cm above the proximal joint surface, the angle between the axes and the distance between the anatomical and mechanical axes on the proximal joint axis, a mean correction angle of the MC I of 22.60° (SD 2.53°) was identified (again, this means the correction angle which would be necessary to align the AA and MA). Since an osteotomy level of 1 cm above the proximal joint line often cannot be precisely achieved, the correction angle was also calculated for an osteotomy height of 1.5 cm above the joint line. The mean correction angle at 1.5 cm above the joint line was 26.73° (SD 2.55°) (Table 5).

Up to now, a common approach to calculate the correction angle of an upcoming Wilson osteotomy has been to determine the angle necessary to achieve 90° between the AA and PA. This approach was also calculated for all healthy individuals and resulted in an average correction angle of only 10.31° (SD 3.12°). This led to an average angle of 3.85° (SD 0.83°) between the AA and MA (Table 6).

### 3.4. Comparison of 3D and 2D Data

The difference between the average measured angles in 2D and 3D was relatively small: 0.18° for the AA-MA angle, 0.28° for MA-PA and 0.60° for AA-PA (Appendix B).

### 3.5. Intraobserver Study

The values for absolute and relative intraobserver variability for the measured angles and lengths are highlighted in Table 7. ICC (intraclass correlation coefficient) values of close to one indicate high reliability (Table 7).

## 4. Discussion

Although some research about the measurement of MC I axes can be found, there is no common definition. Rusli et al. used statistical shape modeling to investigate the variability between the first metacarpal bones of 50 cadaveric specimens using CT data. They found a mean first metacarpal tilt angle of 88.5°, with a range of 18.6°, suggesting that this parameter varies substantially within the population [2]. The average first metacarpal tilt angle (anatomically) found in healthy volunteers in the present study is 79.35° (SD 3.10°), with a smaller variation than the population of Rusli et al. Miura et al. conducted kinematic analysis in vivo on lateral fluoroscopy images of fourteen healthy and eight symptomatic hands with CMC I OA [3]. They were the first to find a significantly greater average volar tilt (=smaller metacarpal tilt angle) in the patient group (73° ± −1°) than in the two control groups (79° ± −1° (<50 yrs) and 82° ± 2° (>50 yrs)). By dividing load transmission on the metacarpal joint facet into an axial and dorsal vector, they hypothesized that excessive volar tilt increases the dorsal vector of the load, which attenuates ligament strength with age and results in dorsal translation of the center of rotation. They concluded that this predisposition could explain the efficacy of MC I extension osteotomy as treatment of early-stage OA [2,3]. These findings were confirmed by Kurosawa et al., who examined lateral X-rays of 132 hands and divided them into three groups according to their severity of CMC I OA: the mean facet angle (=volar tilt) was 83° (±3°) in the healthy hand group, 80° (±4°) in the group with mild OA and 74° (±−5°) in the group with severe OA. Within the group of OA patients, they also found a significant moderately positive correlation between dorsal subluxation and facet angle [4]. By evaluating thumb radiographs of 108 women without CMC I joint symptoms and conducting stress radiographs, Kim et al. found that a steeper metacarpal slope (=greater volar tilt of the first metacarpal) is significantly associated with increased joint laxity. They hypothesized a greater volar tilt to be a risk factor for CMC I OA and emphasized the need of a longitudinal study with follow-up of asymptomatic subjects [5]. The average value of the healthy volunteers in our population did not differ from the average first metacarpal tilt angle of the six symptomatic OA patients (78.88° with SD 3.53°). A potential reason for this circumstance might be that only early-stage OA patients are eligible for Wilson osteotomy and were therefore included in this study. This indicates a multifactorial genesis of CMC I osteoarthritis, and sole corrective osteotomy probably does not fully address the problem. Therefore, most authors, including us, combine Wilson osteotomy with a stabilizing ligamentoplasty.

In the present study, we propose a system for the determination of MC I axes from CT data which is reproducible. The proximal joint axis (PA) was defined with reference to the literature but with inclusion of three-dimensionality. Two different longitudinal axes were determined. The anatomical axis (AA), which is described in the literature, corresponds to the inertia axis and is approximately parallel to the dorsal cortex [2,3,4,5]. However, this axis only focuses on the shape of the bone and leaves out biomechanics. The mechanical axis (MA), which takes the two adjacent joints into account and thus the guidance of the force, was added. The subdivision of the longitudinal axis into an anatomical and a mechanical axis is essential for the determination of the correction angle for Wilson osteotomy. The sole consideration of the anatomical axis is not sufficient to explain the effect of Wilson osteotomy, since the aim of this procedure is to re-center the proximal part of the MC I over the trapezium in order to direct the force to the healthier part of the joint [9,16].

**Figure 11:**
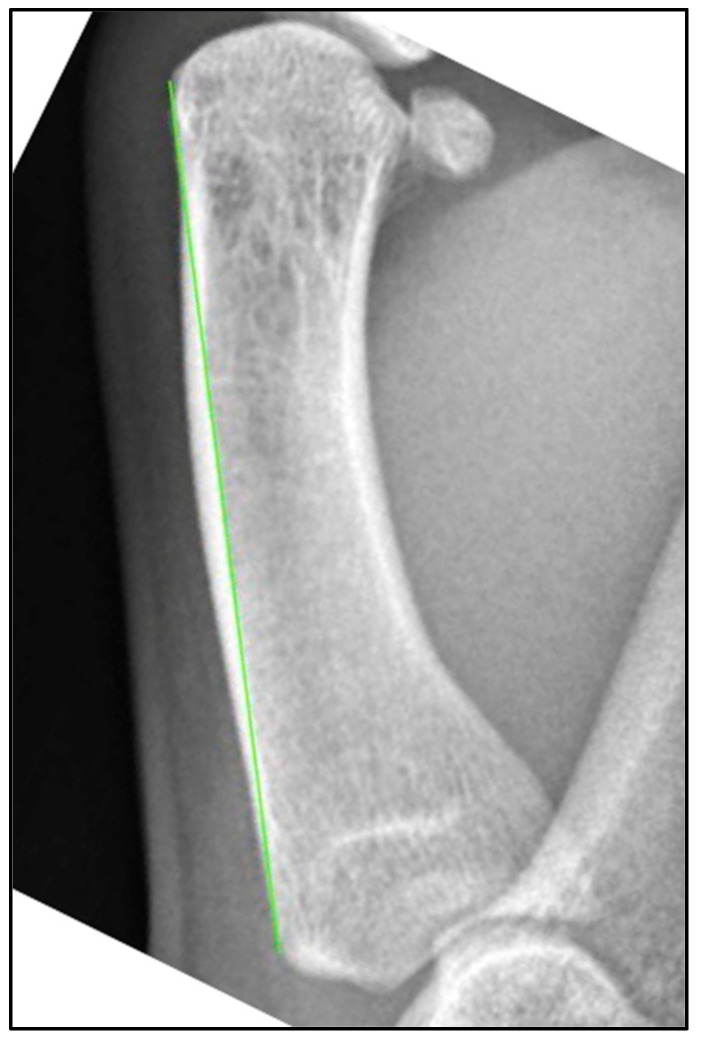
Lateral radiograph of a first metacarpal bone showing uneven dorsal cortex shape.

The comparison of the average values of 2D and 3D data showed that even with 2D data, the above-mentioned axes can be determined relatively reliably, and the Wilson osteotomy correction angle can be calculated. This could explain why, up to now, Wilson osteotomy has led to good results in patients even with no or little planning based on 2D radiographs. Due to the small sample size, this small difference cannot be statistically validated as insignificant. Probably, there is no strong necessity of a 3D calculation in standard cases; especially in the absence of adequate infrastructure or when minimizing radiation exposure is the aim, a 2D approach might be sufficient. However, the measurement methods described in the literature need to be adapted so that there is an anatomical and mechanical component, and it must be taken into account that the shape of the dorsal cortex may vary (Figure 11). Anyhow, 3D planning might be a very useful tool for a more precise calculation, automated planning, robotic-assisted surgery and as a basis for 4D-CT examinations or extraordinary (revision) cases.

The calculated average Wilson osteotomy correction angle of almost 22.6° at 1 cm distal to the CMC I joint aligns the anatomical and mechanical axis.

Taking into account two different osteotomy heights (1 cm and 1.5 cm) results in a range for the correction angle which corresponds to the range of correction described in the literature [6,7,8,9]. This range depends on the shape of the bone, the osteotomy level and the angles between the axes of the MC I. To perform the correction correctly, we recommend patient-specific calculation for each correction.

**Figure 12:**
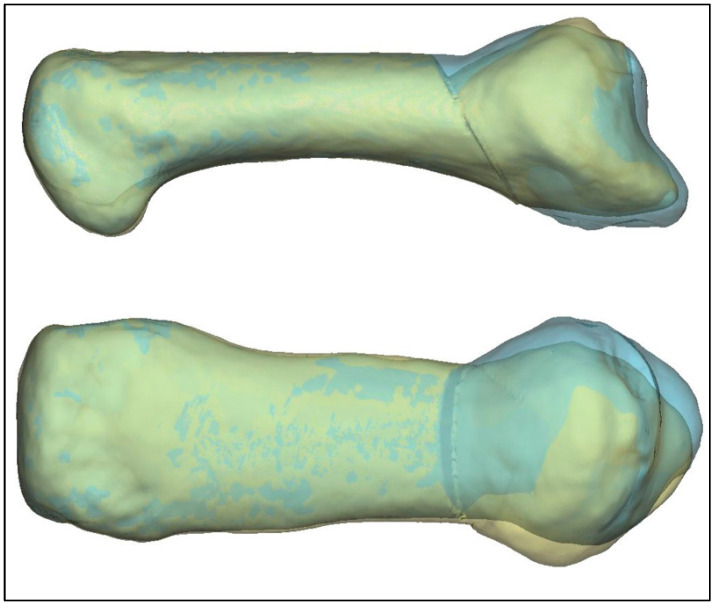
Difference between calculated correction of MC I (yellow) and postoperative situation (blue).

The comparison of the pre- and postoperative datasets showed that the anatomical and mechanical axes approach each other, although the alignment is not perfect. Figure 12 shows the differences between the calculated (22.6°) and executed (28.7°) correction in two projections, highlighting a slight over-correction in the actually conducted osteotomies (Figure 12). The analyzed osteotomies were planned using 2D data with an alternate angle method. Due to the few available postoperative data, no conclusive statement can be made about the reason for this difference, but the surgical technique might be one possible reason. The comparison of the retrospectively calculated and actually performed osteotomies show that the surgical technique can be improved with an accurately calculated correction angle.

The osteotomy simulation with the aim of a 90° metacarpal tilt angle (anatomically) showed that that this is not a sufficient correction. The anatomical and mechanical axes are still at an angle to each other after this correction. Based on the symptomatic preoperative data, it is clear that a larger correction angle is required to achieve the goal of Wilson osteotomy, which is, in our perspective, an alignment of the anatomical and mechanical axes. This is confirmed by the literature, where a correction angle significantly larger than 10° is mostly recommended.

With the two different ways of processing the data as an intraobserver study, the reproducibility of the measured angles was investigated. The results showed that our proposed definition of the angles is reproducible and can be determined without prior knowledge. The deviation of the values in the two measurements shows that the mirror plane and the definition of the curve on the proximal MC I joint surface from dorsal to volar (see Figure 6) are most prone to error. Table 7, which shows the absolute and relative intraobserver variability of the measured values, highlights some deviations. The reason for this is the aforementioned alignment of the curve in the proximal articular surface and the sphere on the MC I head, which is not simple to determine in all cases. Nevertheless, the results show that these two factors do not have a major influence on the final measurements of the correction angle.

A limitation to this study is the limited study population size.

## 5. Conclusions

There is a lack of information in the literature about the MC I axes determined in three dimensions. The hereby proposed definition of the three important axes with consideration of the anatomy, biomechanics and their dependence on each other is a new development. Some elements from the literature were taken into account but had to be adapted. The axes defined for the MC I can be used to determine the correction angle of a Wilson osteotomy or to serve as a foundation for other anatomical studies.

If the axes are defined with small adjustments on a lateral 2D radiograph, similar values can be calculated, and a 3D dataset is not necessarily required. However, this approach would need further analysis.

The correction angle of 20–30° determined by Wilson could be reproduced. However, the angle of correction is variable depending on osteotomy height, bone thickness and the angle between the axes.

Due to the incorporation of three-dimensionality and biomechanics, we also consider the hereby proposed axis measurement system to be potentially useful in the development of CMC I prostheses and their ideal placement in the bone.

Further studies are needed on the interaction between the axes of the MC I and the trapezium, as well as on average angles for healthy and symptomatic (correlated with CMC I OA severity) individuals. Especially, longitudinal studies with long-term follow-up of symptomatic individuals with regard to their anatomical and mechanical axes are needed.

## Figures and Tables

**Table 1:** Angle measurement in healthy data.

	Angle AA-MA [°]	Angle MA-PA [°]	Angle AA-PA [°]	Angle AA-MA Dorsal View [°]	Angle PA–Mirror Plane Proximal View [°]
Mean	6.09	85.40	79.35	1.45	9.63
Standard deviation	1.25	2.88	3.18	0.86	5.40

**Table 2:** Angle measurement in symptomatic data (preoperative).

	Angle AA-MA [°]	Angle MA-PA [°]	Angle AA-PA [°]	Angle AA-MA Dorsal View [°]	Angle PA–Mirror Plane Proximal View [°]
Mean	5.95	84.79	78.88	1.26	9.54
Standard deviation	1.15	3.40	3.86	0.49	4.66

**Table 3:** Angle measurement in symptomatic data (postoperative).

	Angle AA-MA [°]	Angle MA-PA [°]	Angle AA-PA [°]	Angle AA-MA Dorsal View [°]	Angle PA–Mirror Plane Proximal View [°]
Mean	1.30	107.14	107.60		
Standard deviation	0.66	3.90	4.51		

**Table 4:** Comparison between calculated and executed correction angles.

Patient Number	Executed Correction Angle [°]	Calculated Correction Angle at 1 cm above the Proximal Joint Surface [°]	Calculated Correction Angle at 1.5 cm above the Proximal Joint Surface [°]
21	27.15	28.82	24.29
22	30.58	31.20	26.37
23	21.38	23.83	20.13
24	35.59	27.97	23.45
Mean	28.68	22.45	26.35
Standard deviation	5.97	3.07	2.60

**Table 5:** Calculated correction angle for the healthy dataset at two heights.

	Calculated Correction Angle at 1 cm above the Proximal Joint Surface [°]	Calculated Correction Angle at 1.5 cm above the Proximal Joint Surface [°]
Mean	22.60	26.73
Standard deviation	2.53	2.55

**Table 6:** Angles for correction osteotomy to achieve a 90° angle between AA and PA.

	Correction Angle [°]	Angle AA-MA [°]	Angle MA-PA [°]	Angle AA-PA [°]
Mean	10.31	3.85	93.23	89.56
Standard deviation	3.12	0.83	1.02	0.71

**Table 7:** Results of the intraobserver study.

Measurement	Absolute Intraobserver Variability				Relative Intraobserver Variability				ICC
	Absolute Difference [°]		Individual Standard Deviation [°]		Absolute Difference [%]		Individual Standard Deviation [%]		
	Mean	Standard Deviation	Mean	Standard Deviation	Mean	Standard Deviation	Mean	Standard Deviation	
Angle AA-MA	0.76	0.50	0.38	0.25	12.45	8.83	6.23	4.42	0.7
Angle MA-PA	0.77	0.55	0.39	0.27	0.91	0.64	0.45	0.32	0.95
Angle AA-PA	0.84	0.51	0.42	0.26	1.05	0.62	0.52	0.31	0.95
Angle AA-MA (dorsal view)	0.77	0.63	0.32	0.32	100.76	75.52	50.38	37.76	0.21

## Data Availability

The original contributions presented in this study are included in the article; further inquiries can be directed to the corresponding author.

## Appendix A. Computed Tomography Acquisition Settings

Scanner	SIEMENS/SOMATOM Force
Pixel size	0.300781 mm
Width	512 px
Height	512 px
Field of View	154.00 mm
Orientation	RAB
Slice increment	0.3 mm
Slice thickness	0.6 mm

## Appendix B. Comparison of Mean 2D and 3D Data

	**AA-MA [°]**	**MA-PA [°]**	**AA-PA [°]**	**Calculated Correction Angle 1 cm above the Proximal Joint Surface [°]**	**Calculated Correction Angle 1.5 cm above the Proximal Joint Surface [°]**
Absolute difference in mean 2-D and 3-D data	0.18	0.28	0.39	0.60	0.18
Absolute difference in standard deviation 2-D and 3-D data	0.15	0.18	0.20	1.16	1.65
